# Supplementary material for: Isoniazid Concentration and NAT2 Genotype Predict Risk of Systemic Drug Reactions during 3HP for LTBI
Source: J Clin Med. 2019 Jun 6;8(6):812. doi: 10.3390/jcm8060812 (PMC6616849; doi:10.3390/jcm8060812)
Supplement: Supplementary file 1 [file jcm-08-00812-s001.pdf]

Supplement File

Methods

1. Measurement of serum drug concentrations

1.1. Liquid Chromatography–Electrospray Ionisation Mass Spectrometry (LC-ESI/MS)

LC separations were performed using an Agilent 1290 UPLC system equipped with a binary solvent pump, autosampler, sample reservoir, and column oven. An Agilent ZORBAX Eclipse Plus C18 2.1 × 100 mm^2 (1.8 μm) column (Agilent Technologies, Waldbronn, Germany) was employed for the separation. The mobile phase was composed of 5 mM ammonium acetate (solvent A) and 0.1% formic acid in 90% acetonitrile (solvent B) with a flow rate of 0.4 mL/min. A linear gradient was used from 1% to 95% solvent B within the first 6 min. Isocratic elution with 95% solvent B was maintained for 6–7 min. The sample reservoir and column oven were maintained at 4 °C and room temperature (25 °C), respectively. The injection volume was 5 μL.

The mass spectrometric analysis was performed using an Agilent 6460 triple quadrupole system (Agilent Technologies). The positive electrospray ionisation mode was employed with the following parameters: dry gas temperature of 350 °C, dry gas flow rate of 11 L/min, nebuliser pressure of 50 psi, sheath gas temperature of 350 °C, sheath gas flow rate of 11 L/min, nozzle voltage of 0 V, and capillary voltage of 3500 V. The multiple reaction monitoring transition pairs and the collision energy (CE) of the four target compounds (isoniazid, acetyl-isoniazid, rifapentine, desacetyl-rifapentine) and their four isotopes' internal standards (isoniazid-d4, acetyl-isoniazid-d4, rifapentine-d8, desacetyl-rifapentine-D8) are listed in **Table S1**.

1.2. Plasma Sample Preparation Procedure

Blood samples were placed on ice immediately after collection, and plasma was separated through centrifugation within 1 hour of blood collection, after which it was stored at −80 °C until analysis in the core laboratory of National Taiwan University. Plasma samples collected at other hospitals were preserved on dry ice during transport to the laboratory.

To process the plasma samples, protein precipitation was performed by mixing 100 μL of a plasma sample with 300 μL of methanol containing internal standard. The mixture was vortexed using a Geno/Grinder 2010 (SPEX, Metuchen, NJ, USA) at 1000 rpm for 2 minutes. The sample was then centrifuged at 15,000× *g* for 10 minutes at 4 °C, and 100 μL of the supernatant was transferred into an Eppendorf containing 100 μL of deionised water. Finally, the resultant solution was filtered through a 0.22 μm cellulose membrane and subjected to LC-ESI/MS analysis.

1.3. Validation

The optimised chromatogram is shown in **Figure S1**. A calibration curve was generated for each analyte using six concentration levels and the coefficient of determination was larger than 0.996 for four calibration curves. The accuracy was evaluated at three concentration levels and the recoveries were within 100±15%. The intraday precision and intermediate precision were within 2% relative standard deviation, except for concentrations lower than limit of quantification (200 ng/mL).

2. Genotyping of Drug-Metabolising Enzymes

Genomic DNA was extracted from peripheral blood using the Quick-DNA Plus kit (Zymo Research) according to the manufacturer's protocol. Polymorphisms of *N-acetyltransferase 2* (*NAT2*), *cytochrome P450 2E1* (*CYP2E1*), and *arylacetamide deacetylase* (*AADAC*) were identified from the National Center for Biotechnology Information dbSNP database (www.ncbi.nlm.nih.gov/SNP). The SNPs were genotyped using the MassARRAY system (Sequenom, San Diego, CA, USA), and the

primer extension products were analysed using matrix-assisted laser desorption/ionisation time-of-flight mass spectrometry as previously described [1,2]. Details of the primers used are listed in **Table S2**.

Table S1. Retention time (Rt) and mass parameters of four target compounds and their isotopes' internal standards.

Compound Name	Formula	Rt (min)	CE (V)	Precursor Ion (m/z)	Production ion Q1 (m/z)	Production ion Q2 (m/z)
Isoniazid	$C_6H_7N_3O$	1.557	20	138.0	121.0	79.0
Acetyl-isoniazid	$C_8H_9N_3O_2$	1.525	20	180.0	121.2	138.1
Rifapentine	$C_{47}H_{64}N_4O_{12}$	5.378	20	877.3	845.2	453.2
Desacetyl-rifapentine	$C_{45}H_{62}N_4O_{11}$	4.824	20	835.2	803.3	153.1
Isoniazid-d4	$C_6H_3D_4N_3O$	1.555	20	142.1	125.1	83.1
Acetyl-isoniazid-d4	$C_8H_5D_4N_3O_2$	1.523	20	184.1	125.1	142.2
Rifapentine-d8	$C_{47}H_{56}D_8N_4O_{12}$	5.348	40	885.5	151.1	95.1
Desacetyl-rifapentine-d8	$C_{45}H_{54}D_8N_4O_{11}$	4.794	20	843.5	811.3	95.1

Table S2. Primer sequences.

Gene & SNP ID	PCR-Forward Primer	PCR-Reverse Primer	Extension Primer	Allele 1 (mass)	Allele 2 (mass)
NAT2 rs1208	ACGTTGGATGTTTGGGCACGAGATTTCTCC	ACGTTGGATGAACTCTCACTGAGGAAGAGG	GAGGTTGAAGAAGTGCTGA	A (6228.1)	G (6244.1)
NAT2 rs1041983	ACGTTGGATGCCATGCCAGTGCTGTATTTG	ACGTTGGATGCAGACCACAATGTTAGGAGG	AGCAATGTTAGGAGGGTATTTTTA	C (7709.1)	T (7789)
NAT2 rs1799929	ACGTTGGATGGGCAGGAGATGAGAATTAAG	ACGTTGGATGTGCTTGACAGAAGAGAGAGG	TATCTCCTGATTTGGTCCA	T (6016)	C (6032)
NAT2 rs1799930	ACGTTGGATGACGTCTGCAGGTATGTATTC	ACGTTGGATGCCTGCCAAAGAAGAAACACC	CATAGACTCAAAATCTTCAATTGTT	G (7846.2)	A (7926.1)
NAT2 rs1799931	ACGTTGGATGGGGTGATACATACACAAGGG	ACGTTGGATGAAATCTCGTGCCCAAACCTG	GTTCCTTATTCTAAATAGTAAGGGAT	G (8246.4)	A (8326.3)
NAT2 rs1801279	ACGTTGGATGCCATGGAGTTGGGCTTAGAG	ACGTTGGATGTTGATTGACCTGGAGACACC	TTGATCACATTGTAAGAAGAAACC	A (7640)	G (7656)
NAT2 rs1801280	ACGTTGGATGGACCCAGCATCGACAATGTA	ACGTTGGATGCAAATACAGCACTGGCATGG	TGTAATTCCTGCCGTCA	T (5407.6)	C (5423.6)
CYP2E1 rs2031920	ACGTTGGATGGTTCTTAATTCATAGGTTGC	ACGTTGGATGTCATTTCTCATCATATTTTC	TTAATTCATAGGTTGCAATTTT	T (7000.6)	C (7016.6)
CYP2E1 rs2070673	ACGTTGGATGACATCCAGGAACATGTTGCC	ACGTTGGATGTTGTCTAACCAGTGCCAAAG	AGTTAAGAACGTGGGGTGAGGTACCG	T (8411.5)	A (8467.4)
CYP2E1 rs3813867	ACGTTGGATGTTGGTTGTGCTGCACCTAAC	ACGTTGGATGAAACCAGAGGGAAGCAAAGG	GGGATTCTTGGTTCAGGAGAG	C (6804.4)	G (6844.5)
CYP2E1 rs6413432	ACGTTGGATGGTGCCAGGATTACAGGTATG	ACGTTGGATGACCACCACACCCAGCTGATT	ACCCAGCTGATTAAAAATT	A (6051)	T (6106.9)
AADAC rs1803155	ACGTTGGATGATAAACCTCTCAGGGAGCAG	ACGTTGGATGCCAGACAACATGTACCTGTG	GAGCAGGGAACTCCAATTAA	G (6406.2)	A (6486.1)
AADAC rs61733692	ACGTTGGATGGCAAATTTTCTGAGGTTTTC	ACGTTGGATGGTATATTGAGTGGCTAAAGG	TATTGAGTGGCTAAAGGAAAATCTA	C (8000.3)	T (8080.2)

AADAC: arylacetamide deacetylase; CYP: cytochrome P450; NAT: N-acetyltransferase; SNP: single nucleotide polymorphism.

Table S3. Association of *NAT2*/*CYP2E1*/*AADAC* single nucleotide polymorphism (those with p value > 0.05) and systemic drug reaction.

		Unadjusted OR (95% CI)	*p* Value	Adjusted OR (95% CI)*	*p* Value
**Additive model**					
NAT2 rs1208	GG	Ref		Ref	
	AA	>99.9 (<0.01->99.9)	0.993	>99.9 (<0.01->99.9)	0.994
NAT2 rs1799929	TT	Ref		Ref	
	CC	1.02 (0.11-9.27)	0.989	0.77 (0.07-8.59)	0.832
NAT2 rs1799930	GG	Ref		Ref	
	GA	3.95 (0.63-24.7)	0.924	1.82 (0.49-6.78)	0.965
	AA	2.11 (0.59-7.55)	0.237	3.48 (0.53-23.0)	0.276
NAT2 rs1799931	GG	Ref		Ref	
	GA	1.42 (0.41-4.99)	0.469	1.20 (0.33-4.33)	0.426
	AA	5.56 (0.93-33.2)	0.086	4.52 (0.70-29.2)	0.130
NAT2 rs1801279	GG	Ref		Ref	
	GA	3.14 (0.57-17.2)	0.980	4.33 (0.70-26.9)	0.978
NAT2 rs1801280	CC	Ref		Ref	
	TT	>99.9 (<0.01->99.9)	0.982	>99.9 (<0.01->99.9)	0.982
CYP2E1 rs2031920	CC	Ref		Ref	
	TT	>99.9 (<0.01->99.9)	0.953	>99.9 (<0.01->99.9)	0.952
CYP2E1 rs3813867	CC	Ref		Ref	
	GG	1.86 (0.23-15.1)	0.961	1.52 (0.18-12.7)	0.702
CYP2E1 rs6413432	TT	Ref		Ref	
	TA	<0.01 (<0.01->99.9)	0.958	<0.01 (<0.01->99.9)	0.957
	AA	2.47 (0.26-23.4)	0.958	2.33 (0.24-22.6)	0.953
AADAC rs1803155	GG	Ref		Ref	
	GA	1.25 (0.23-6.84)	0.902	1.25 (0.22-7.02)	0.959
	AA	1.34 (0.26-7.03)	0.767	1.47 (0.27-8.06)	0.664
AADAC rs61733692	TT	Ref		Ref	
	CC	>99.9 (<0.01->99.9)	0.947	>99.9 (<0.01->99.9)	0.872
**Dominant model**					
NAT2 rs1799930	GG	Ref		Ref	
	AA+GA	2.35 (0.69-7.99)	0.171	2.04 (0.58-7.20)	0.269
NAT2 rs1799931	GG	Ref		Ref	
	GA+AA	1.89 (0.62-5.77)	0.262	1.59 (0.51-4.98)	0.428
CYP2E1 rs6413432	TT	Ref		Ref	
	AA+AT	0.54 (0.07-4.44)	0.564	0.55 (0.06-4.77)	0.591
AADAC rs1803155	GG	Ref		Ref	
	AA+GA	1.30 (0.27-6.17)	0.744	1.36 (0.28-6.64)	0.704
**Recessive model**					
NAT2 rs1799930	GG+GA	Ref		Ref	
	AA	2.63 (0.50-13.7)	0.252	2.49 (0.45-13.6)	0.295
NAT2 rs1799931	GG+GA	Ref		Ref	
	AA	5.00 (0.88-28.6)	0.070	4.27 (0.69-26.4)	0.118
CYP2E1 rs6413432	TT+AT	Ref		Ref	
	AA	2.87 (0.30-27.2)	0.359	2.64 (0.27-25.7)	0.404
AADAC rs1803155	GG+GA	Ref		Ref	
	AA	1.15 (0.37-3.58)	0.813	1.26 (0.39-4.01)	0.702

* Adjusted for age, sex and estimated glomerular filtration rate.

Table S4. Plasma concentration of isoniazid (INH) and rifapentine (RPT) stratified by age**.

	C6			C24		
	**Age >50 (*n*= 22)**	**Age 30 ~ 50 (*n* = 17)**	**Age <30 (*n* = 13)**	**Age >50 (n = 43)**	**Age 30 ~ 50 (*n* = 24)**	**Age <30 (*n* = 16)**
INH (ug/mL)	2.40 [1.13–3.71]	1.27 [0.87–5.71]	1.93 [1.29–3.36]	0.10 [0.05–0.23]	0.05 [0.04–0.08]*	0.05 [0.05–0.13]*
RPT (ug/mL)	21.3 [16.7–32.9]	21.9 [19.4–26.9]	18.7 [15.1–29.6]	12.2 [8.20–16.3]	10.4 [8.04–13.0]	11.4 [8.77–14.0]

Data are median [1st quartile – 3rd quartile]. * $P < 0.05$ comparing with age >50 by using Kruskal–Wallis test. **One could have multiple samples and all samples were included in the analysis.

Table S5. Plasma concentration of isoniazid (INH), acetyl-isoniazid (AcINH), rifapentine (RPT), and desacetyl-rifapentine (DeAcRPT) stratified by estimated glomerular filtration rate (eGFR)*.

	C6				C24			
	**eGFR ≥90 (n=28)**	**eGFR 60 ~ 90 (n=22)**	**eGFR <60 (n=2)**	***p* Value**	**eGFR ≥90 (n=52)**	**eGFR 60 ~ 90 (n=27)**	**eGFR <60 (n=4)**	***p* Value**
INH (ug/mL)	1.72 [1.14 – 3.51]	2.54 [1.19 – 4.53]	1.57 [1.53 – 2.28]	0.686	0.05 [0.04 – 0.13]	0.07 [0.05 – 0.39]	0.11 [0.10 – 0.16]	0.045
Ac-INH (ug/mL)	7.63 [4.90 – 9.99]	10.01 [7.55 – 13.16]	14.81 [12.48 – 16.94]	0.007	0.35 [0.24 – 0.60]	0.68 [0.44 – 0.93]	1.81 [1.64 – 2.92]	<0.0001
RPT (ug/mL)	20.23 [16.59 – 28.9]	23.23 [17.06 – 33.16]	32.28 [21.61 – 38.00]	0.449	11.39 [7.96 – 13.99]	12.03 [8.81 – 15.34]	15.21 [12.92 – 17.29]	0.026
DeAcRPT (ug/mL)	6.88 [5.19 – 9.45]	8.32 [5.84 – 10.09]	7.55 [6.41 – 12.2]	0.435	11.13 [7.64 – 13.98]	11.08 [9.51 – 14.21]	18.98 [15.71 – 19.23]	0.030

Data are median [1st quartile – 3rd quartile]. *p* values were calculated using the Kruskal–Wallis test. * One could have multiple samples and all samples were included in the analysis.

Table S6. Comparison of plasma concentration of isoniazid (INH), acetyl-isoniazid (AcINH), rifapentine (RPT), and desacetyl-rifapentine (DeAcRPT) for first and second months.

	C6 (*n* = 32)			C24 (*n* = 57)		
	**1st Month**	**2nd Month**	***p* Value**	**1st Month**	**2nd Month**	***p* Value**
INH (ug/mL)	1.27 [1.08–2.77]	1.69 [1.02–3.30]	0.837	0.05 [0.05–0.14]	0.06 [0.05–0.12]	0.641
AcINH (ug/mL)	8.88 [6.09–12.71]	9.15 [7.32–10.96]	0.082	0.44 [0.26–0.80]	0.43 [0.29–0.79]	0.709
RPT (ug/mL)	21.04 [16.65–36.14]	21.11 [16.90–30.02]	0.501	10.81 [7.94–13.43]	10.97 [7.96–14.58]	0.829
DeAcRPT (ug/mL)	7.68 [5.29–10.36]	7.62 [5.34–8.95]	0.350	11.14 [7.79–13.98]	11.01 [7.90–12.71]	0.835

Data are median [1st quartile – 3rd quartile]. *p* values were calculated using the Wilcoxon rank-sum test. One could have multiple samples and all samples were included in the analysis.

Table S7. Literature review of original reports (**S7A**) and case reports (**S7B**) for flu-like syndrome suspected due to a rifamycin.

S7A.

Total cases	TB status	Incident cases (%)	HIV	RMP Dosage	Concomitant drugs	Onset after starting Tx	Duration of symptoms
49 [3]	Active TB	8 (16%)	NA	1200 mg BIW	INH, pyridoxine	2.5–4 hr	24 hr
115 [4]	Active TB	9 (8%)	NA	900 mg BIW	INH	NA	NA
119 [4]	Active TB	5 (4%)	NA	600 mg BIW	INH	NA	NA
115 [4]	Active TB	25 (22%)	NA	900 mg QW	INH	NA	NA
117 [4]	Active TB	12 (10%)	NA	600 mg QW	INH	NA	NA
116 [5]	Active TB	32 (55%)	NA	900–1200 mg QW (22.1-24.4 mg/kg)	EMB, PZA	NA	NA
94 [5]	Active TB	23 (24%)	NA	900 mg QW (21.5 mg/kg)	INH	NA	NA
96 [5]	Active TB	11 (11%)	NA	600 mg QW (13.7 mg/kg)	INH	NA	NA
68 [6]	Active TB	15 (22%)	NA	900-1200 mg BIW	EMB	1~6 M: 13%; 6~12 M: 7%; 13~18 M: 1%	NA
72 [6]	Active TB	36 (50%)	NA	900-1200 mg QW	EMB	1~6 M: 26%; 6~12 M: 24%	NA
77 [6]	Active TB	31 (40%)	NA	450 mg/day, then 900-1200 mg QW	EMB	1~6 M: 12%; 6~12 M: 18%; 13~18 M: 10%	NA
288 [7]	Active TB	2 (0.7%)	NA	600 mg BIW	INH	NA	NA
530 [8]	Active TB	2 (0.3%)	NA	600 mg/day	INH, EMB	Mean: 30 days	NA
8 [9]	RA	1 (12.5%)	NA	600 mg/day 8 wks, then 900 mg/day 8 wks, then 1200 mg/day	NA	4th weeks	NA
2868 [10]	Active TB	12 (0.4%)	NA	600 mg/day	INH, EMB, PZA	Mean: 36 days	NA
667 [11]	Leprosy	54 (8.1%)	NA	600 mg/day	DDS, CFZ	NA	NA
3893 [12]	LTBI	87 (2.2%)	negative	600–900 mg QW	INH	Usually at 3rd dose	< 24 hours
207 [13]	LTBI	2 (1%)	positive	600–900 mg QW	INH	Usually at 3rd dose	< 24 hours

Ab: antibody; BIW: twice weekly; CFZ: clofazimine; DDS: diaminodiphenyl sulfone; EMB: ethambutol; HIV: human immunodeficiency virus; INH: isoniazid; NA: not available; PZA: pyrazinamide; QW: once weekly; RA: rheumatic arthritis; RMP: rifampin; TB: tuberculosis; Tx: treatment.

S7B.

Age (yr)/Sex	Disease	HIV	RMP Dosage	Concomitant drugs	Onset time	Duration of symptoms
55/F [14]	Leprosy	NA	600 mg monthly	CFZ, DDS	10th dose	24 hrs
30/F [15]	Leprosy	NA	600 mg monthly	CFZ, DDS	1st dose	< 12hrs
35/F [15]	Leprosy	NA	600 mg monthly	CFZ, DDS	2nd dose	< 12hrs
33/M [16]	Leprosy	NA	600 mg monthly	DDS	3rd dose	2 days
22/M [17]	Leprosy	NA	600 mg monthly	CFZ, DDS	27th dose	24 hrs
18/M [18]	Leprosy	NA	600 mg monthly	CFZ, DDS	2nd dose	5-6 days
45/M [19]	Leprosy	NA	600 mg monthly	CFZ, DDS	2nd dose	24 hrs
40/F [19]	Leprosy	NA	450 mg monthly	CFZ, DDS, PZA	4th dose	NA
25/M [19]	Leprosy	NA	600 mg monthly	CFZ, DDS	9th dose	72 hrs
64/M [20]	Leprosy	NA	450 mg monthly	CFZ	3th dose	< 12 hrs
42/F [21]	Leprosy	NA	150 mg*	NA	2nd dose	24 hrs

CFZ: clofazimine; DDS: diaminodiphenyl sulfone; HIV: human immunodeficiency virus; NA: not available; PZA: pyrazinamide; RMP: rifampin; * Unknown rifampin dosing and frequency before 1st dose of rifabutin 150mg rechallenge. For each case of leprosy before receiving monthly pulse RMP treatment, RMP 600mg daily dose combined with DDS and CFZ were given for several months.

Table S8. Literature review of original reports (**S8A**) and case reports (**S8B**) for flu-like syndrome due to isoniazid (INH) proven by re-challenge.

S8A.

Total cases	TB status	Incident cases (%)	Mean age (yr)	Sex	HIV	INH Dosage	Concomitant drugs	Mean onset after starting Tx (days)	Duration of symptoms
112 [22]	Active TB	11 (9.8)	NA	NA	NA	300 mg/day (15 mg/kg/day)	RMP	NA	NA
814 [23]	Active TB	8 (1.0)	66	M: 5F: 3	negative	300 mg/day	RMP	21 days	12 hrs

HIV: human immunodeficiency virus; INH: isoniazid; NA: not available; RMP: rifampin; TB: tuberculosis; Tx: treatment.

S8B

Age (yr) /Sex	TB status	HIV	INH Dosage	Concomitant drugs	Onset after starting Tx	Symptoms/Signs	Duration of symptoms
62/F [24]	Active TB	NA	100 mg BID	none	14 days	Fever, tachycardia, malaise, back and leg pain	2 days
57/F [25]	LTBI	NA	300 mg/day	none	9 days	Fever, malaise, respiratory distress, hypotension	10 hrs
54/F [25]	LTBI	NA	300 mg/day	none	8 days	Fever, chills	NA
53/M [26]	LTBI		300 mg/day	none	12 days	Fever, chills, nausea, fatigue	NA
48/M [27]	Active TB	NA	300 mg/day	RMP/PZA	4 days	Fever, chills, erythematous maculopapular rash, nausea	48 hrs

56/F [28]	Active TB	NA	300 mg/day	pyridoxine	14 days	Fever, nausea, vomiting, hypotension, confusion	24 hrs
84/F [29]	Active TB	NA	300 mg/day	RMP	14 days	Fever, rigor, confusion, hypotension	24 hrs
64/F [29]	Active TB	NA	600 mg BIW	RMP/PZA BIW	8 days	Fever, rigors, rash	NA
68/F [29]	Active TB	NA	300 mg/day	RMP/PZA	8 days	Fever, rigors,	NA
10/F [30]	Active TB	negative	100 mg/day (4.6 mg/kg/day)	RMP/EMB/PZA	6 days	Fever, chill, rhinorrhea, dry cough, nausea, body ache	12 hrs

BID: twice daily; BIW: twice weekly; EMB: ethambutol; HIV: human immunodeficiency virus; NA: not available; PZA: pyrazinamide; RMP: rifampin; TB: tuberculosis; Tx: treatment.

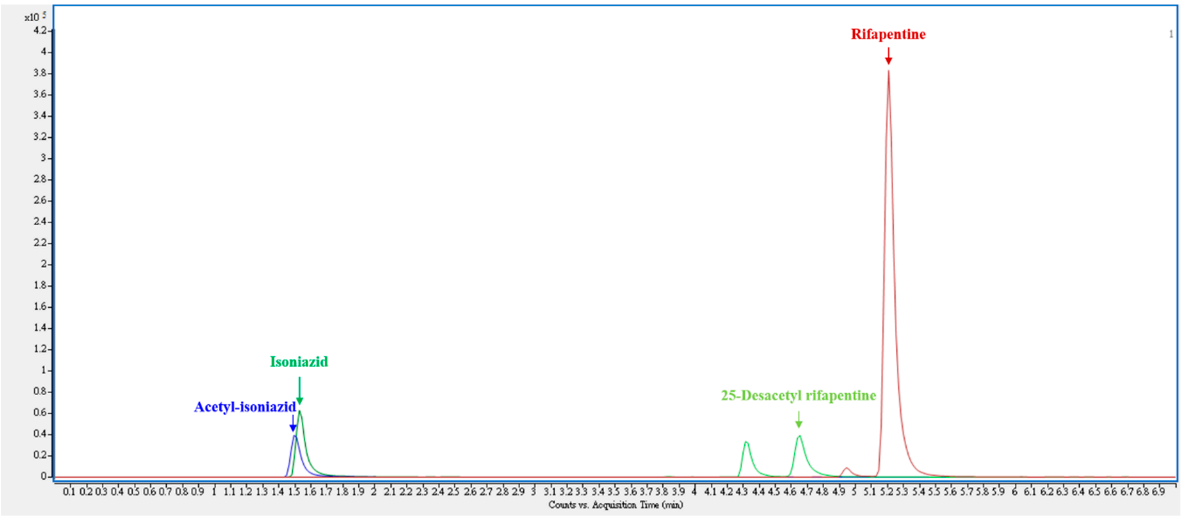

Figure S1. The multiple reaction monitoring chromatogram of isoniazid, rifapentine and their metabolites in standard spiked plasma. Concentration of isoniazid, acetyl-isoniazid and rifapentine was 0.5 μg/mL; concentration of desacetyl-rifapentine was 10 μg/mL.

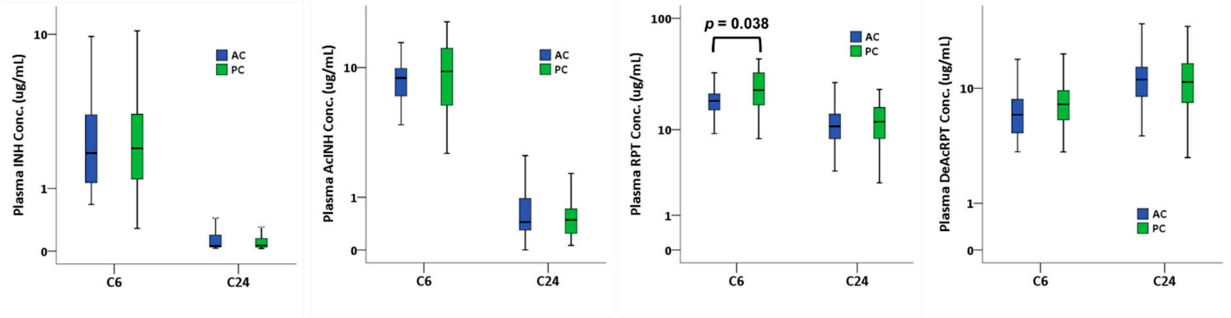

Figure S2. Boxplot showing the plasma concentration of isoniazid (INH), acetyl-isoniazid (AcINH), rifapentine (RPT), and desacetyl-rifapentine (DeAcRPT) at 6 (C6) and 24 (C24) hours after dosing, stratified by the timing of taking medication, either before (AC) or after meal (PC) (p value calculated using the Mann–Whitney U test).

References

1 Jurinke, C.; van den Boom, D.; Cantor, C.R.; Koster, H. The use of MassARRAY technology for high throughput genotyping. *Adv. Biochem. Eng. Biotechnol.* **2002,** 77, 57-74.

2 Jurinke, C.; van den Boom, D.; Cantor, C.R.; Koster, H. Automated genotyping using the DNA MassArray technology. *Methods Mol. Biol.* **2002**, *187*, 179-192.

3 Poole, G.; Stradling, P.; Worlledge, S. Potentially serious side effects of high-dose twice-weekly rifampicin. *Br. Med. J.* **1971**, *3*, 343-347.

4 Controlled trial of intermittent regimens of rifampicin plus isoniazid for pulmonary tuberculosis in Singapore. *Lancet* **1975**, *2*, 1105-1109.

5 Dickinson, J.M.; Mitchison, D.A.; Lee, S.K.; Ong, Y.Y.; O'Mahoney, M.G.; Girling, D.J.; Nunn, A.J. Serum rifampicin concentration related to dose size and to the incidence of the 'flu' syndrome during intermittent rifampicin administration. *J. Antimicrob. Chemother.* **1977,** *3*, 445-452.

6 A controlled trial of daily and intermittent rifampicin plus ethambutol in the retreatment of patients with pulmonary tuberculosis: results up to 30 months. *Tubercle* **1975,** *56*, 179-189.

7 Dutt, A.K.; Jones, L.; Stead, W.W. Short-course chemotherapy of tuberculosis with largely twice-weekly isoniazid-rifampin. *Chest* **1979,** *75*, 441-447.

8 Zierski, M.; Bek, E. Side-effects of drug regimens used in short-course chemotherapy for pulmonary tuberculosis. A controlled clinical study. *Tubercle* **1980**, *61*, 41-49.

9 Gabriel, S.E.; Conn, D.L.; Luthra, H. Rifampin therapy in rheumatoid arthritis. *J. Rheumatol.* **1990,** *17*, 163-

166.
10 Chien, J.Y.; Chien, S.T.; Huang, S.Y.; Yu, C.J. Safety of rifabutin replacing rifampicin in the treatment of tuberculosis: a single-centre retrospective cohort study. *J. Antimicrob. Chemother.* **2014,** *69*, 790-796.
11 Brasil, M.T.; Opromolla, D.V.; Marzliak, M.L.; Nogueira, W. Results of a surveillance system for adverse effects in leprosy's WHO/MDT. *Int. J. Lepr. Other Mycobact. Dis.* **1996,** 64, 97-104.
12 Sterling, T.R.; Moro, R.N.; Borisov, A.S.; Phillips, E.; Shepherd, G.; Adkinson, N.F.; Weis, S.; Ho, C.; Villarino,M.E.; Tuberculosis Trials Consortium. Flu-like and Other Systemic Drug Reactions Among Persons Receiving Weekly Rifapentine Plus Isoniazid or Daily Isoniazid for Treatment of Latent Tuberculosis Infection in the PREVENT Tuberculosis Study. *Clin. Infect. Dis.* **2015**, *61*, 527-535.
13 Sterling, T.R.; Scott, N.A.; Miro, J.M.; Calvet, G.; La Rosa, A.; Infante, R.; Chen, M.P.; Benator, D.A.; Gordin, F.; Benson, C.A.; et al. Three months of weekly rifapentine and isoniazid for treatment of Mycobacterium tuberculosis infection in HIV-coinfected persons. *AIDS* **2016,** *30*, 1607-1615.
14 Naafs, B.; Matemera, B.O. A possible 'flu' syndrome on once-monthly rifampicin. *Lepr. Rev.* **1986**, *57*, 271-272.
15 Patki, A.H.; Jadhav, V.H.; Mehta, J.M. 'Flu' syndrome on once monthly rifampicin. *Indian J. Lepr.* **1988,** *60*, 84-86.
16 Parking, A.A.; Shah, B.H. Flu like syndrome with rifampicin pulse therapy. *Indian J. Lepr.* **1989,** *61*, 209-210.
17 al-Samie, A.R.; al-Qubati, Y. "Flu" syndrome on monthly rifampin dose; first case reported from Yemen. *Int. J. Lepr. Other Mycobact. Dis.* **1995,** *63*, 574-576.
18 Salafia, A.; Candida. Rifampicin induced flu-syndrome and toxic psychosis. *Indian J. Lepr.* **1992,** *64*, 537-539.
19 Thomas, A.; Joseph, P.; Prabhakar, R. "Flu" syndrome associated with other systemic manifestations with once a month rifampicin in the treatment of multibacillary leprosy. *Indian J. Lepr.* **1993,** *65*, 219-224.
20 Namisato, M.; Ogawa, H. Serious side effects of rifampin on the course of WHO/MDT: a case report. *Int. J. Lepr. Other Mycobact. Dis.* **2000,** *68*, 277-282.
21 Choong, K.; Looke, D. Cross-reaction to rifabutin after rifampicin induced flu-like syndrome and thrombocytopenia. *Scand. J. Infect. Dis.* **2011,** *43*, 238.
22 Eule, H.; Werner, E.; Winsel, K.; Iwainsky, H. Intermittent chemotherapy of pulmonary tuberculosis using rifampicin and isoniazid for primary treatment: the influence of various factors on the frequency of side-effects. *Tubercle* **1974,** *55*, 81-89.
23 Dutt, A.K.; Moers, D.; Stead, W.W. Undesirable side effects of isoniazid and rifampin in largely twice-weekly short-course chemotherapy for tuberculosis. *Am. Rev. Respir. Dis.* **1983**, *128*, 419-424.
24 Krasnitz, A. Drug fever due to administration of isoniazid. *Am. Rev. Tuberc.* **1953**, *68*, 249-252.
25 Davis, R.S.; Stoler, B.S. Febrile reactions to INH. *N. Engl. J. Med.* **1977,** *297*, 337.
26 Jacobs, N.F. Jr.; Thompson, S.E. 3rd. Spiking fever from isoniazid simulating a septic process. *JAMA* **1977,** *238*, 1759-1760.
27 Henderson, R.P.; Davis, H.L.; Self, T.H. Spiking fever induced by isoniazid. *Drug Intell. Clin. Pharm.* **1983,** *17*, 741-742.
28 Gabrail, N.Y. Severe febrile reaction to isoniazid. *Chest* **1987,** 91, 620-621.
29 Motion, S.; Humphries, M.J.; Gabriel, S.M. Severe 'flu'-like symptoms due to isoniazid--a report of three cases. *Tubercle* **1989,** 70, 57-60.
30 Pandit, S.; Choudhury, S.; Das, A.; Datta, S.; Das, S.K. Isoniazid-induced flu-like syndrome: A rare side effect. *Lung India* **2013,** *30,* 61-63.
